# A new variety of *Didymocarpus* (Gesneriaceae) from Guangdong, China

**DOI:** 10.3897/phytokeys.128.35446

**Published:** 2019-07-23

**Authors:** Wen-Jing Xu, Wei-Hua Qin*, Zi-Qi Wang, Zhong-Lin Li, Long-Fei Fu, Xin Hong

**Affiliations:** 1 Anhui Provincial Engineering Laboratory of Wetland Ecosystem Protection and Restoration, School of Resources and Environmental Engineering, Anhui University, Hefei 230601, Anhui, China Anhui University Hefei China; 2 Nanjing Institute of Environmental Sciences, Ministry of Ecology and Environment of the People’s Republic of China, CN–210042, Nanjing, Jiangsu Province, China Nanjing Institute of Environmental Sciences Nanjing China; 3 Guangxi Key Laboratory of Plant Conservation and Restoration Ecology in Karst Terrain, Guangxi Institute of Botany, Guangxi Zhuang Autonomous Region and Chinese Academy of Sciences, CN-541006, Guilin, Guangxi Zhuang Autonomous Region, China Guangxi Institute of Botany, Guangxi Zhuang Autonomous Region and Chinese Academy of Sciences Guilin China; 4 The Gesneriad Conservation Center of China, Guilin Botanical Garden, Chinese Academy of Sciences, CN-541006, Guilin, Guangxi Zhuang Autonomous Region, China Guilin Botanical Garden, Chinese Academy of Sciences Guilin China

**Keywords:** New variety, *
Didymocarpus
*, Gesneriaceae, China

## Abstract

A new variety of *Didymocarpus*, D.heucherifoliusvar.gamosepalus from Guangdong, China, is described and illustrated with photographs. It closely resembles the more widespread *D.heucherifolius* within a number of morphological characters. However, it can be easily distinguished from the latter according to the new taxon: calyx base connate, 5-lobed from middle to above middle, larger flowers (up to 5 cm long) and glabrous corolla.

## Introduction

*Didymocarpus* Wall. is comprised of 31 species in China at present. W.T. Wang examined the genus *Didymocarpuss.l.* and divided them into two Sections: Section Didymocarpus (herbs with stems) and Section Heteroboea (herbs without stems) W.T. Wang auct. non Benth. Section Heteroboea is regarded as a distinct group, characterised by a rosulate habit and having a thick rootstock (Burtt 1998; Weber and Burtt 1998). This Section has varied considerably over time, due to the difficulty in using molecular phylogenetic studies and morphological revisions. A few more species, from China, were recently transferred to *Petrocodon* Hance (Weber et al. 2011). *Didymocarpuscortusifolius* and *D.heucherifolius* were treated as species of *Chirita* Buch.-Ham. ex D. Don. (Weber et al. 2000), but *Chirita* was cancelled in 2011 (Wang et al. 2011, Weber et al. 2011). However, they still belong to the Section Didymocarpus before any further research is undertaken.

In March 2019, several *Didymocarpus* specimens without flowers were collected by the authors during field investigations in Guangdong province. The plant at ﬁrst looked like *D.heucherifolius* because of its similar leaf shape and leaf hair morphology. Subsequently, the living plants were cultivated in the nursery of the Gesneriad Conservation Center of China (GCCC). After we observed and collected specimens with flowers, we were surprised to ﬁnd that the flower structures of the two species were different. Measurements and morphological character assessments of the putative species were undertaken and described using the living material in the GCCC. All morphological characters were studied under dissecting microscopes and are described using the terminology presented by Wang et al. (1998).

## Taxonomic treatment

### Didymocarpus heucherifolius Handel-Mazzetti var. gamosepalus

Taxon classificationPlantaeLamialesGesneriaceae

X.Hong & F.Wen sp. nov.

cda51327-0998-458a-8763-8c810d458f11

urn:lsid:ipni.org:names:77200427-1

Fig. 1

#### Diagnosis.

Didymocarpusheucherifoliusvar.gamosepalus can be distinguished from D.heucherifoliusvar.heucherifolius by its calyx base connate, 5-lobed from middle to above middle, glabrous corolla ca. 5 cm long, stamens 2.3 cm from the base, staminodes 1.4–1.8 cm from the base and 3.6–4.3 cm pistil. It also can be distinguished from *D.heucherifoliusvar.yinzhengii* by its calyx base connate, 5-lobed from middle to above middle, stamens 2.3 cm from the base, staminodes 1, pistil 3.6–4.3 cm.

#### Type.

CHINA. Guangxi Province, cultivated in the nursery of Gesneriad Conservation Center of China (GCCC), introduced from north of Guangdong Province: Pingyuan County, Meizhou City, growing in rocky crevices at the foot of a calcareous sedimentary rocky hill. 22 February 2019, flowering, *WF20190222-05* (holotype: IBK; isotype: AHU)

#### Description.

Acaulescent perennial herb. Rhizome horizontal, 3–4 cm long, up to 1.5 cm thick, roots fibrous. Leaves 4–8 basal, clustered at the apex of the rhizome; clearly whorled, orbicular-ovate to triangular, 3–9 × 3.5–11 cm, papery, base cordate, apex rounded, margin irregularly triangular denticulate, upper surface densely covered with eglandular short hairs and sparse long hairs, lower surface sparsely covered with short and long hairs confined to the veins; basal veins 4 or 5, lateral veins 3–4 on each side of midrib, palmate; petioles terete, 2–9.5 cm long, densely covered with fuscous hairs. Cymes 1–4, axillary, 4 to many flowered; peduncle 10–18 cm long, densely covered with brown villous, pedicel 1–2.5 cm long, with same indumentum as on the peduncle. Bracts 2, opposite, subulate to subulate-triangular, ca. 6 mm long, adaxially glabrous, abaxially puberulent, margin sparsely denticulate, densely ciliary villous; bracteoles 2, opposite, subulate, 2–3 mm long, indumentum same as bracts. Calyx actinomorphic, 6–6.5 mm long, shallowly 5-lobed to about two-thirds of the calyx length from the base, lobes equal, ca. 2 × 1.5 mm, apices obtuse, margin sparsely denticulate, inside glabrous, outside white puberulent. Corolla zygomorphic, up to 5 cm; glabrous both inside and outside, pink to magenta, inside with two brightly yellow strips at throat. Tube funnel-shaped to tubular, 1.8–2.2 cm long, inflated in the middle, orifice 1–1.5 cm in diameter, base constricted; limb distinctly 2-lipped, adaxial lip 2-lobed to near middle, ca. 0.6–0.8 × 1.6 cm, obliquely triangular, abaxial lip 3-lobed to base, lobes rounded or oblong, ca. 1.1 × 1.1 cm, more or less equal. Stamens 2, adnate to corolla ca. 2.3 cm above the corolla base; filaments 8–10 mm long, straight, swollen at middle, white, glabrous with glandules on the surface; anthers ca. 2 mm long, white bearded. Staminodes 3, adnate to 1.4–1.8 cm above base of corolla tube, 0.3–0.5 mm long, white, glabrous. Disc annular, ca. 1 mm high. Pistil 3.6–4.3 cm long, densely puberulent; ovary white, ca. 3.3–4 cm long, cylindrical, puberulent; style ca. 3 mm long; stigma 1, terminal, depressed-globose, centrally sunken, undivided, translucent. Capsule purplish-red when young, linear-cylindrical, puberulent, up to 9 cm.

**Figure 1.**
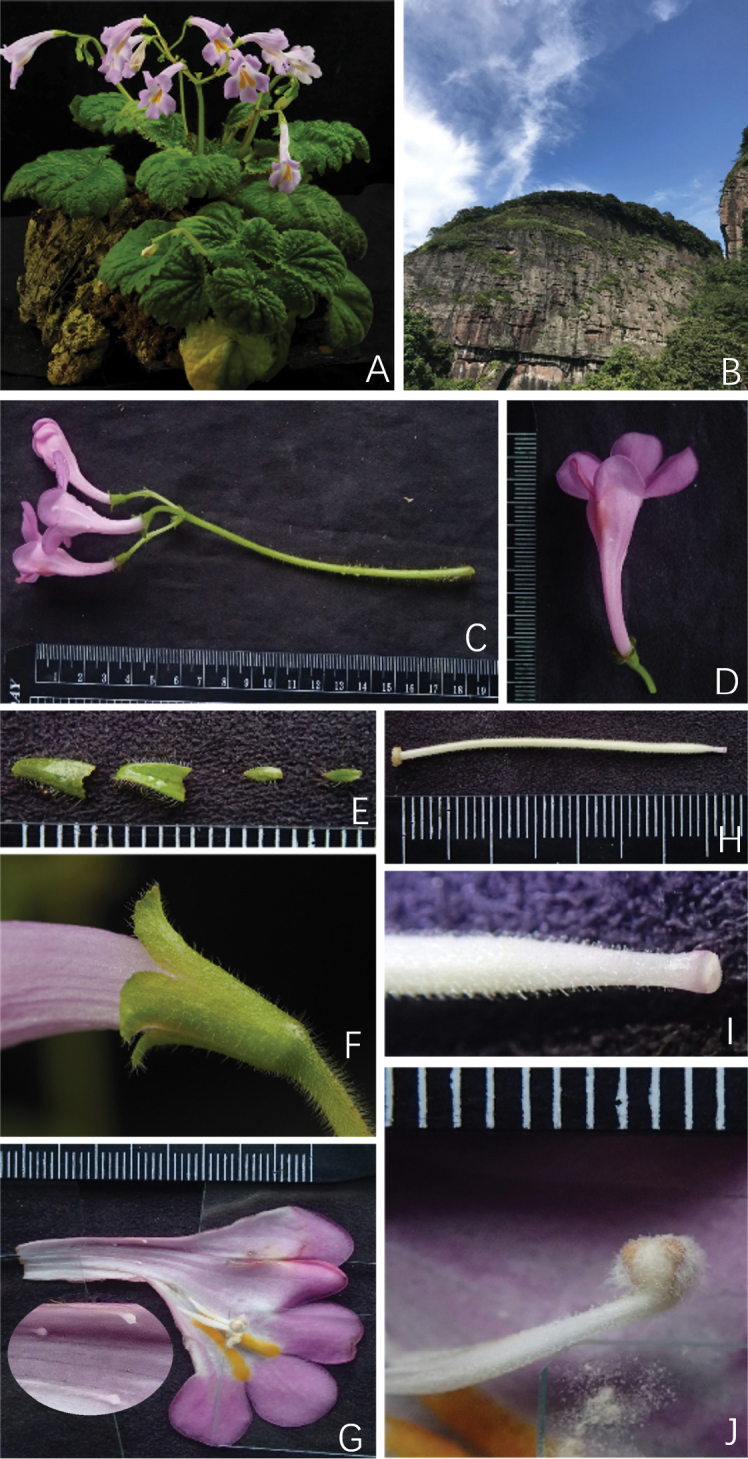
Didymocarpusheucherifoliusvar.gamosepalus**A** habit **B** habitat, showing Danxia landscape **C** cyme with flowers **D** corolla, showing outside glabrous **E** bracts and bracteoles **F** calyx, showing base of calyx connate **G** opened corolla, showing staminodes **H** pistil **I** stigma **J** stamens.

#### Etymology.

The specific epithet is derived from calyx 5-lobed from middle to above middle.

#### Vernacular name.

Hé è Mín Gàn Cháng Shùo Jù Tái (Chinese pronunciation); 合萼闽赣长蒴苣苔 (Chinese name).

#### Distribution and habitat.

The new variety has so far been found only in the type locality, near Pingyuan County, Meizhou City, Guangdong Province. The landform of the type locality is Danxia landform, which is formed from red-coloured sandstones and conglomerates deposited by sedimentation from lakes and streams from mainly the Cretaceous age. The new variety is locally abundant and grows on moist and shaded rocky faces on the cliff in subtropical evergreen seasonal rain forest. The average temperature of Pingyuan County is about 21.7 °C and the average annual precipitation is over 1 600 mm. Flowering is from February to March.

#### Notes.

Didymocarpusheucherifoliusvar.gamosepalus and the type variety, D.heucherifoliusvar.heucherifolius, share a number of similar vegetative characters, but the new variety differs from the latter in several morphological features, such as larger flowers and glabrous corolla, calyx base connate, 5-lobed from middle to above middle. A detailed comparison of the diagnostic characters between Didymocarpusheucherifoliusvar.gamosepalus and other variety of *D.heucherifolius* is shown in Table 1.

**Figure 2.**
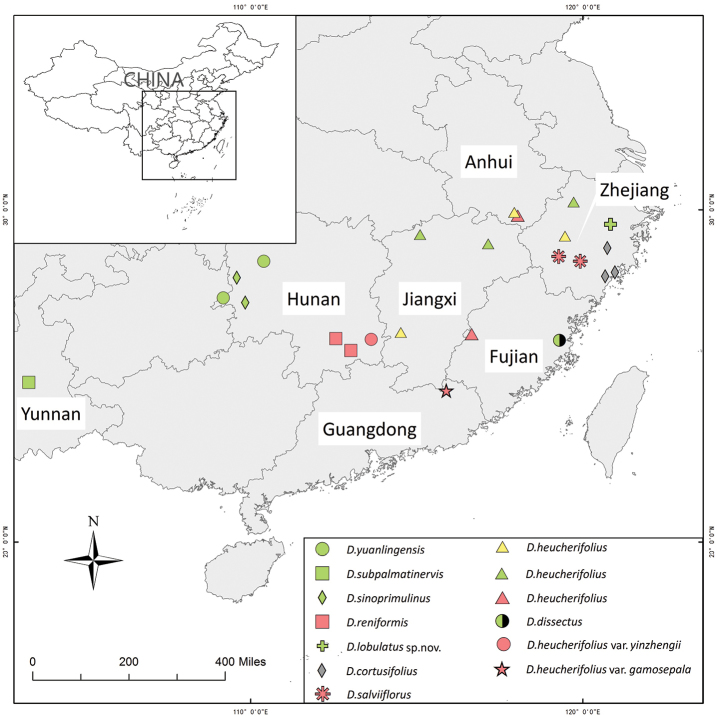
The distribution areas of Section Heteroboea and their Parent material. The different colours represent the different Parent material: red – danxia landscape; yellow – granite landscape; green – karst limestone landscape; nlack – volcanic landscape).

There are nine species and two varieties in DidymocarpusSectionHeteroboea, including an unpublished new species: *D.lobulatus* sp. nov. These species are mostly distributed in Eastern China, of which, more than 50% are distributed in the Zhejiang province (shown in Figure 2). The northernmost species is the *D.heucherifolius* in Linan County, Hangzhou City, Zhejiang Province, while D.heucherifoliusvar.gamosepalus is the southernmost species. *D.heucherifolius* is the most widespread species, which can be found in Danxia, Karst limestone and Granite landscapes. As shown in Figure 2, nine localities are Karst landscape (the green points) and nine localities are Danxia landscape (the red points). The majority of Section Heteroboea species (four species and two varieties) were reported on Danxia landscape.

**Table 1.** Comparison of the diagnostic characters of Didymocarpusheucherifoliusvar.gamosepalus and other variety of *D.heucherifolius*.

**Characters**	** D. h. var. heucherifolius **	*** D. h. var. yinzhengii ***	** D. h. var. gamosepalus **
Shape of calyx	shallowly 5-lobed to the base, lobes unequal	shallowly 5-lobed to the base, lobes unequal	base connate, 5-lobed from middle to above middle, lobes equal
Size of corolla	2.5–3.2 cm long	up to 4 cm	ca. 5 cm long
Indumentum of corolla	puberulent	glabrous	glabrous
Stamens	1.0–1.2 cm from the base	1.0–1.2 cm from the base	2.3 cm from the base
Staminodes	0.6–0.8 cm from the base	absent	1.4–1.8 cm from the base
Pistil size	1.8–2.9 cm	up to 3 cm	3.6–4.3 cm

## Supplementary Material

XML Treatment for Didymocarpus heucherifolius Handel-Mazzetti var. gamosepalus
